# Editorial: Placebo and Nocebo Effects in Psychiatry and Beyond

**DOI:** 10.3389/fpsyt.2020.00801

**Published:** 2020-08-07

**Authors:** Katja Weimer, Paul Enck, Seetal Dodd, Luana Colloca

**Affiliations:** ^1^Department of Psychosomatic Medicine and Psychotherapy, Ulm University Medical Center, Ulm, Germany; ^2^Department of Psychosomatic Medicine and Psychotherapy, University Hospital Tübingen, Tübingen, Germany; ^3^The Institute for Mental and Physical Health and Clinical Translation, Deakin University, Geelong, VIC, Australia; ^4^Centre for Youth Mental Health, University of Melbourne, Parkville, VIC, Australia; ^5^Department of Psychiatry, University of Melbourne, Parkville, VIC, Australia; ^6^University Hospital Geelong, Barwon Health, Geelong, VIC, Australia; ^7^Department of Pain and Translational Symptom Science, School of Nursing, University of Maryland, Baltimore, MD, United States; ^8^Departments of Anesthesiology and Psychiatry, School of Medicine, University of Maryland, Baltimore, MD, United States; ^9^Center to Advance Chronic Pain Research, University of Maryland, Baltimore, MD, United States

**Keywords:** placebo effect, nocebo effect, learning, expectancy, conditioning, psychotherapy, psychiatry

## Introduction

The placebo effect is part of every medical intervention and plays a crucial role in randomized placebo-controlled trials (RCTs). It is beneficial to maximize the placebo effect when treating patients, but it should be minimized in RCTs to estimate the true drug effect (1). Studies have shown that the placebo effect is formed by learning mechanisms (2), and an expert consensus has suggested that the beneficial effects of placebo can be harnessed for clinical use to improve patient outcomes (3). In contrast to the placebo effect, adverse events can occur and symptoms can get worse through a negative placebo effect, the so-called nocebo effect (4). Yet, to exploit placebo mechanisms in clinical practice a lot of questions remain unanswered. For this Research Topic Issue, we called for the latest research articles in the field of placebo and nocebo research. The issue comprises 38 articles from “Hypothesis and Theory” to “Reviews” and to “Original Research” articles.

After giving an overview about the underlying mechanisms of the placebo effect, such as conditioning, expectations and influencing factors, Friesen summarizes ethical views regarding the use of the placebo effect. Until recently, it has been assumed that placebos take only effect when patients are deceived, but she encourages considering placebos as a “source of agency”, without deception and in agreement with patients’ autonomy. Babel complements the current view about classical conditioning in the placebo effect. In fact, many studies use a combination of classical conditioning and verbal suggestions to induce placebo and nocebo effects. Due to recent studies using hidden and subliminal conditioning procedures, Babel argues that classical conditioning is a distinct mechanism that works without conscious expectations. However, there are only a few studies limited to the area of pain and further studies are needed.

## The Placebo Effect in Psychotherapy

Particularly in psychiatry, patients are not only treated with pharmacotherapy but often with different forms of psychotherapy. The role and mechanisms of the placebo effect in psychotherapy has been repeatedly discussed, and Enck and Zipfel point to the challenges of disentangling specific effects of the different psychotherapeutic approaches including unspecific and the placebo effect. This is even more challenging when considering that many psychotherapeutic approaches are equally effective and there is still a debate within psychotherapy research about the specific, common and unspecific factors (also known as the “Dodo bird verdict”). Enck and Zipfel encourage psychotherapy researchers as well as therapists to understand that the placebo effect exists and provide a framework that acknowledges context, common, and specific factors for further research. With her Mini Review, Blease attempts to provide greater clarity in the definition of the placebo effect in psychotherapy and gives insights into controversial views such as “psychotherapy is a placebo”. She argues that the problem could be solved when placebos and the placebo effect are clearly defined the same way as they are defined in clinical trials: as control interventions and the effect they induce. In the first instance, it seems to be contradictory that Blease recommends using a clear definition of the placebo effect, whereas Jonas states that “the placebo response is a myth” and does not exist. According to his arguments it is contradictory that an inert treatment will produce a response and votes for a broader understanding of this response that should be called “meaning response” or “healing response”. However, these two views are compatible and in line with the definitions of “placebo effect” as the effect elicited by placebo mechanisms, and “placebo response” as all health changes after administration of an inert treatment, as stated by expert consensus of placebo researchers published in 2018 (3).

## The Role of Context Factors in Placebo and Nocebo Effects

In psychotherapy research, context factors such as the patient-provider interaction are considered a common factor, albeit they are considered to be part of the placebo response in other treatments. In their systematic review, Daniali and Flaten found that aspects of a positive patient-provider interaction such as higher confidence in the provider, perceived higher competence and professionalism, and positive nonverbal behaviors were associated with lower pain reports and higher placebo effects in patients and participants. In contrast, negative nonverbal behaviors led to higher pain reports and nocebo effects. Howe et al. delve deeper in specific aspects of the patient-provider-interaction and differentiate between competence and warmth. They provide a framework for researchers and practitioners about how patients perceive competence and develop the feeling that the physician “gets it”, and how they perceive warmth when the physician “gets them”. However, non-specific effects of treatments comprise many aspects, and Gerger et al. translated and validated the first German version of the Healing Encounters and Attitudes Lists (HEAL-D) and its short form (HEAL-D-SF). This set of questionnaires assesses patients’ views on the patient-provider interaction, the healthcare environment, treatment expectations, positive outlook, spirituality, and attitudes towards complementary and alternative medicine. It may help to turn non-specific into specific effects, and therefore may be usable for research purposes and clinical practice.

To evaluate how and how often oncologists make use of empathy expressions by practitioners, van Vliet et al. assessed video-taped consultations between oncologists and patients with advanced breast cancer in an observational study. Overall, oncologists often provided information about expectancy and used several empathic behaviors such as understanding, respecting, supporting and exploring, whereas a lack of empathy was less often observed. Further studies should evaluate effects of empathic expressions on treatment outcomes and (nocebo) side effects. Not only physicians are aware of the effect of unspecific factors on treatments, patients are aware of them, too: In their large online survey among Italian patients with musculoskeletal pain, Rossettini et al. found that patients believe that contextual factors such as an empathetic alliance, and verbal and non-verbal communication are effective and work through mind-body connections. Furthermore, they have positive attitudes towards their use in clinical practice if they are not used in a deceptive way.

One of the challenges in placebo research is to disentangle the placebo effect from other effects through elaborate study designs. To differentiate the placebo effect from the psychosocial context, Gruszka et al. as well as Curkovic et al. recommend outsourcing some parts of the psychosocial context *via* smartphone applications. Such an app could be used for standardized recruitment, randomization and the provision of treatment information to induce positive expectations. Furthermore, it could be used to assess expectations, symptom severity, or physiological data *via* smartphone sensors (e.g., heart rate) without personal interaction and in daily life. Additionally, Curkovic et al. suggest that studies should rigorously investigate and report aspects of research plans to the better investigate which aspects of an intervention at which dose is relieving symptoms, and this could also be achieved through an app.

## The Placebo Effect on Depression, Anxiety, Pain, and Other Symptoms

Irving Kirsch published several studies and meta-analyses about the placebo response and placebo effect in treatments with antidepressants and questioned whether the placebo response and the drug effect in RCTs are additive (5, 6). In his recent article, Kirsch summarizes the results of these and other meta-analyses clearly demonstrating that “most (if not all) of the benefits of antidepressants in the treatment of depression and anxiety are due to the placebo response”. However, RCTs cannot answer the question how patients’ symptoms evolve without any treatment or how they should be treated instead. Kirsch reports several alternative treatments such as psychotherapy, physical exercise, omega-3 supplements, and yoga that has been shown to be as effective as antidepressants but with less side effects, and in some cases with better long-term effects than antidepressants. To further evaluate how expectancy could influence outcomes in antidepressant trials, Laferton et al. performed a re-analysis of a double-blind RCT in major depression comparing escitalopram, S-adenosyl-L-methionine (SAMe) and placebo. Results show that the patients’ perceived treatment assignment during the trial changed, was predicted by symptom improvements, and contributed more to treatment outcomes than actual treatment. Finally, there was no difference between groups.

But patients do not only “feel better” through the placebo effect, several neuroimaging studies could demonstrate neurophysiological changes in the brain. Brown and Pecina underline these results and provide an overview of neuroimaging studies of the antidepressant placebo effect. They show that this effect is comparable to the placebo effect on pain. This finding implies common underlying mechanisms involving brain areas associated with cognitive control, the representation of expectations, and reward and emotional processes.

Still, pain is the best investigated symptom in placebo research. Complementary to neuroimaging studies, Reicherts et al. present an electroencephalography (EEG) study combining the motivational priming hypothesis and the conditioning of placebo and nocebo effects. Participants who were told that unpleasant pictures decrease pain, indeed reported less pain, and consequently, somatosensory evoked potentials were decreased when they watched unpleasant pictures compared to neutral pictures. They conclude that the well-known modulation of pain by emotions is influenced by expectations.

The experimental pain study by Zhou et al. found interactional effects of different expectations, sex of participants and personal characteristics such as dispositional optimism and state anxiety on pain reports in a complex manner. After a conditioning procedure with electrical pain, women in the low expectancy group reported decreased pain compared to the No or High expectancy groups, whereas men reported decreased pain in the High expectancy group in the test session. Whether optimism or state anxiety predicted placebo effects was dependent on the expectancy level, but independent of sex. To explore other predictors of placebo analgesia, Wang et al. used latent class analyses (LCA) to identify learning patterns during a conditioning procedure in an experimental pain study. LCA revealed that greater or increased differences between high and low pain ratings in combination with red and green light signaling stimuli during conditioning were associated with greater placebo analgesia in the subsequent testing phase. Furthermore, expectations of pain decrease were a mediator for placebo analgesia, but higher age and higher warmth-detection thresholds were associated with lesser placebo analgesia.

A large proportion of our knowledge about the placebo effect and its underlying mechanisms stems from experimental studies with pain, but there is little knowledge whether the same mechanisms apply to other symptoms. To elucidate this question, Wolters et al. reviewed the literature about placebo and nocebo effects in dyspnea, fatigue, nausea, and itch. They can confirm that in general the same mechanisms as in pain are at work in these symptoms, such as the combination of verbal suggestions and conditioning, and that subjective symptoms are more prone to elicit a placebo effect than are physiological measures. However, there are also some differences as the influence of individual characteristics varies between symptoms. Evidence can be added by an experimental study by Meeuwis et al. who investigated placebo and nocebo effects through verbal suggestions on itch. Participants received the respective information either in an open-label condition knowing that the applied tonic was a placebo (a pink-colored skin disinfectant), or in a closed-label condition in which they were deceptively told that the tonic was effective. Whereas suggestions did not affect itch reports during histamine iontophoresis, participants in both positive suggestion groups reported lower itch and lower skin temperature increase after the iontophoresis compared to the negative suggestion groups. Interestingly, their open-label suggestion was as effective as the deceptive information about the effectiveness of the placebo, and they found a symptom specific physiological reaction to itch.

Another underreported areas are placebo and nocebo effects on cardiac symptoms and physiology. In an experimental study with patients with Takotsubo cardiomyopathy—a rare, reversible form of cardiomyopathy after stressful psychosocial life events—and heart-healthy controls, all participants received a saline infusion three times together with the information that it has no effect, a positive (placebo) or negative (nocebo) effect on cardiac functions, respectively. Olliges et al. report that before and during the nocebo condition subjective stress rating, heart rate, and systolic blood pressure increased, whereas the latter also increased after placebo information. However, there were no differences between patients and controls.

## Areas Related to Mental Disorders

The placebo effect could not only be helpful to directly decrease symptoms of a disorder, but also when it is used to influence functions related to mental disorders such as cognitive functioning or appetite regulation. Participants in the study of Fuhr and Werle were randomized to listen to a mental training or philosophy lecture both audio-taped for 20 min, and half of the participants of each group were told that they listen to an effective or control tape. All participants improved their cognitive performance as measured with a d2-test, but those participants who experienced a greater improvement rated the received treatment as effective irrespective of group assignment. This, at least, shows that healthy persons can rate their cognitive performance without being influenced by (bogus) verbal suggestions, and thus, could be indicative of a healthy function. Winkler and Hermann chose a different study design: two groups received a nasal spray along with the suggestion of a cognitive improvement (placebo) or impairment (nocebo) effect, and one group served as a control (without nasal spray or suggestions). Similar to the study by Fuhr and Werle, verbal suggestions did not affect actual cognitive performance. However, participants in the placebo group rated their cognitive improvement better and felt less tired compared to the nocebo group. The authors conclude that these subjective effects may explain why so-called neuroenhancers are still popular among college students. For their study about placebo and nocebo effects of a sham transcranial magnetic stimulation (sTMS), Höfler et al. employed women who turned out to be placebo or nocebo responders, respectively, in previous studies. According to their responsiveness they received the information that the sTMS will increase (placebo) or decrease (nocebo) their left-sided visual attention in an eye-tracking experiment. As in the above-mentioned studies, the placebo instruction did not affect actual visual attention, but subjectively improved attention. In contrast, nocebo responders showed the opposite to the expected reaction.

In another eye-tracking study from the same work group, Potthoff et al. did not directly target visual attention, but a placebo pill that claimed to reduce appetite was given to healthy, mostly normal-weight women, and their reactivity to food cues was registered. Participants reported decreased appetite which was related to decreased visual attention for food, e.g., fixation and dwell time on high and low-caloric food images compared to non-food pictures. The experimental study by Hoffmann et al. confirms these results: healthy normal-weight participants reported decreased appetite after ingesting a placebo pill that should increase satiety compared to a control group. They additionally assessed an objective marker of hunger and found that the opposite information—that a placebo pill claimed to enhance appetite - increased plasma ghrelin levels but did not affect appetite itself. In a third study of placebo effects on food consumption, Panayotov showed that the information about a calorie-reduced diet decreased body mass, body mass index (BMI), and fat tissue in overweight and obese participants of a weight loss program. Although participants did not strictly adhere to their diet programs and the sample size was small, this preliminary study shows that weight regulation could be directly addressed through manipulating expectations of patients.

## Nocebo Effects

In conjunction with studies about the placebo effect, the nocebo effect has already been mentioned above. Previous studies about the “bad brother” of the placebo effect have shown that known placebo mechanisms such as conditioning, expectations, and social learning can also have negative outcomes. Faasse et al. define “nocebo effects as unpleasant or adverse outcomes triggered by the treatment context”. The authors differentiate between primary nocebo effects and nocebo side effects, and the misattribution of regular symptoms to an (inert) treatment. Furthermore, they describe how experimental studies should be designed to investigate the nocebo effect appropriately. While Faasse et al. focus on studies with treatments involving drugs or medical devices, Locher et al. emphasize that the nocebo effect could also occur in psychotherapy. They provide two examples where a nocebo or nocebo-related effect could evolve: In patients with chronic primary pain or other symptoms without a clear physiological etiology, and in relation to trauma debriefing to prevent post-traumatic stress disorders (PTSD).

To prevent nocebo (side) effects it would be helpful if nocebo responders could be detected in advance. In a re-analysis of experimental endotoxemia studies, Benson and Elsenbruch investigated predictors of the nocebo effect. Nocebo responders, defined as participants in the placebo arms of RCTs who believed they were allocated to the verum arm, reported significantly more physical symptoms but did not differ from non-responders in psychological or physical parameters. Within nocebo responders, physical symptoms correlated with greater state anxiety, negative mood, catastrophizing and neuroticism. Their study demonstrates that it is difficult to predict who will be a nocebo responder, but that perceiving nocebo side effects could affect perceived treatment allocation—another reason why nocebo side effects should be reduced. Webster and Rubin provide a systematic review of RCTs investigating brief psychological interventions to reduce or avoid nocebo side effects in medical treatments. In the 27 studies found, omitting side effect information was most successful to reduce nocebo side effects, whereas other communication strategies such as priming, distraction, and altering the branding of drugs showed mixed effects. De-emphasizing of side effects was not effective. Finally, they discuss that it could be challenging to balance the reduction of nocebo side effects with informed consent. Pan et al. investigated another strategy to reduce nocebo side effects in an experimental study: Participants with weekly headaches received a placebo pill and were randomized to read a bogus medication leaflet only or to read additionally an explanation about the nocebo effect. Two minutes after pill intake, the group that had received the explanation about nocebo reported less nocebo symptoms than the other group. This effect was moderated by baseline symptoms, perceived sensitivity to medicine, and expectations. Furthermore, most participants evaluated the nocebo information as helpful.

## Underreported Research Fields

Most of the articles in this Research Topic deal with the placebo effect and response after typical applications of treatments such as pills or ointments. However, disentangling the true treatment effect from the placebo response and placebo effect is also challenging in other forms of treatments, e.g., psychotherapy (see above). Chae et al. discuss in particular two aspects that could lead to a high placebo response in acupuncture: the fact that even sham acupuncture may elicit physiological responses, and the difficulty of effective blinding of provider and patient. They suggest more appropriate alternative control strategies in acupuncture treatment.

There is less research about the placebo effect in children (7) and this Research Topic comprises only two further articles about it: one involved an experimental design with healthy children, and one discusses the influence of the so-called placebo-by-proxy effect. The placebo-by-proxy effect was introduced by Grelotti and Kaptchuk (8) in 2011 and describes the effect where people in the social environment of a patient (parents, siblings, relatives, peers) feel better when the patient receives an effective treatment. Czerniak et al. complement this concept with the corresponding “nocebo-by-proxy” effect and discusses the impact of these two concepts particularly on children’s symptoms and treatments. Their review of the available literature opens an important research field. The influence of parents or other proxies on placebo and nocebo responses has rarely been studied. The experimental study by Watolla et al. investigated the effect of a suggested ginkgo patch on cognitive performance in children and one parent. While they found only a poor overall placebo effect, neither the cognitive performance nor the expectations of children and their parents were interrelated. This may imply that shared information and heritability have a low impact on the placebo effect. Although it should be taken into account that the participants were all healthy and without need for cognitive improvement. This finding is supported by the first study involving a classical twin design: Weimer et al. employed healthy mono- and dizygotic twin pairs in an experimental study with a heat pain paradigm. After conditioning the effectiveness of an ointment, twins reported a significant placebo analgesic effect in the test condition. This effect was mainly related to the personal learning experience during the conditioning procedure, but not to the effect of their co-twin, suggesting that heritability and shared environment play a minor role. In contrast, first studies show a genetic component in the placebo effect, but these results are still inconclusive (9) and twin studies should be combined with genetic analyses to further elucidate this area.

## Maximize or Optimize Treatments Through Placebo Mechanisms

Elsenbruch et al. tie in with first evidence that psychophysiological responses, such as an increase of parasympathetic activation, to placebo interventions could play a role in the establishment of a placebo effect. In their study, a brief progressive muscle relaxation exercise but not a control task reduced heart rate and systolic blood pressure, and decreased pain perceptions in relaxed participants in a pain paradigm with rectal distensions.

Such experimental studies show promising ways to harness the placebo effect for patients’ treatments in ethical and legal ways. Benefits for patients are clear as they experience symptom as well as side effect reductions, but the placebo effect is rarely used systematically. Showing that harnessing the placebo effect is not only effective but also cost-efficient could improve its visibility and acceptability. A systematic review by Hamberger et al. investigated if placebo interventions are also cost-efficient but showed that there is a lack of health economic evaluations and encourage placebo researchers to report costs of placebo interventions.

## Conclusion: More Questions Than Answers?

In summary, the multifaceted articles in this Research Topic issue show that placebo and nocebo effects are complex phenomena. There is still a debate about the role of placebo and nocebo effects in psychotherapy research and their relation to common and context factors. In contrast, context factors such as the patient-provider interaction have already been acknowledged as part of the placebo effect in other treatments. Research about the placebo effect on depression, anxiety, and pain reveals a high placebo effect showing symptom improvement and neurophysiological changes in the brain. However, there is less research about other symptoms such as itch or heart-related diseases, among others. Recent studies aim to harness the placebo effect to improve functions that are related to mental disorders, such as cognitive functioning or appetite regulation, and may be an interesting research area for further studies. There are several other underreported research fields such as: appropriate control conditions for treatments other than pills, placebo and nocebo effects in children, and the role of genetics and heritability. An increasing amount of articles investigate the nocebo effect and nocebo related adverse effects, their mechanisms and strategies to avoid or reduce them. Finally, all research aims to improve treatments of patients and recent studies show promising results by employing techniques that enhance the placebo effect or reduce the nocebo effect. However, more research is needed to transfer knowledge about placebo and nocebo effects into clinical practice to benefit patients in an ethical and broadly accepted manner (10).

## Author Contributions

KW wrote the first draft of the manuscript. PE, SD, and LC provided critical revision of the manuscript and important intellectual contributions. All authors contributed to the article and approved the submitted version.

## Conflict of Interest

The authors declare that the research was conducted in the absence of any commercial or financial relationships that could be construed as a potential conflict of interest.
